# Impact of intraventricular septal fiber orientation on cardiac electromechanical function

**DOI:** 10.1152/ajpheart.00050.2022

**Published:** 2022-03-18

**Authors:** Jairo Rodríguez-Padilla, Argyrios Petras, Julie Magat, Jason Bayer, Yann Bihan-Poudec, Dounia El Hamrani, Girish Ramlugun, Aurel Neic, Christoph M. Augustin, Fanny Vaillant, Marion Constantin, David Benoist, Line Pourtau, Virginie Dubes, Julien Rogier, Louis Labrousse, Olivier Bernus, Bruno Quesson, Michel Haïssaguerre, Matthias Gsell, Gernot Plank, Valéry Ozenne, Edward Vigmond

**Affiliations:** ^1^Inria Epione Team, Université Côte d’Azur, Biot, France; ^2^Johann Radon Institute for Computational and Applied Mathematics, Austrian Academy of Sciences, Linz, Austria; ^3^Liryc, Electrophysiology and Heart Modeling Institute, Fondation Bordeaux Université, Pessac-Bordeaux, France; ^4^Centre de recherche Cardio-Thoracique de Bordeaux, Université Bordeaux, Bordeaux, France; ^5^INSERM, Centre de recherche Cardio-Thoracique de Bordeaux, Bordeaux, France; ^6^Université Bordeaux Bordeaux, Institut de Mathématiques de Bordeaux, UMR 5251, Talence, France; ^7^Centre de Neuroscience Cognitive, CNRS, Université Claude Bernard Lyon I, Villeurbanne, France; ^8^Division of Biophysics, Gottfried Schatz Research Center, Medical University of Graz, Graz, Austria; ^9^BioTechMed-Graz, Graz, Austria; ^10^Cardiology Departement, Bordeaux University Hospital (CHU), Pessac, France; ^11^Centre de Résonance Magnétique des Systèmes Biologiques, CNRS/Université de Bordeaux, Bordeaux, France

**Keywords:** diffusion tensor imaging, electromechanical models, fiber orientation, intraventricular septum, normal structural discontinuities

## Abstract

Cardiac fiber direction is an important factor determining the propagation of electrical activity, as well as the development of mechanical force. In this article, we imaged the ventricles of several species with special attention to the intraventricular septum to determine the functional consequences of septal fiber organization. First, we identified a dual-layer organization of the fiber orientation in the intraventricular septum of ex vivo sheep hearts using diffusion tensor imaging at high field MRI. To expand the scope of the results, we investigated the presence of a similar fiber organization in five mammalian species (rat, canine, pig, sheep, and human) and highlighted the continuity of the layer with the moderator band in large mammalian species. We implemented the measured septal fiber fields in three-dimensional electromechanical computer models to assess the impact of the fiber orientation. The downward fibers produced a diamond activation pattern superficially in the right ventricle. Electromechanically, there was very little change in pressure volume loops although the stress distribution was altered. In conclusion, we clarified that the right ventricular septum has a downwardly directed superficial layer in larger mammalian species, which can have modest effects on stress distribution.

**NEW & NOTEWORTHY** A dual-layer organization of the fiber orientation in the intraventricular septum was identified in ex vivo hearts of large mammals. The RV septum has a downwardly directed superficial layer that is continuous with the moderator band. Electrically, it produced a diamond activation pattern. Electromechanically, little change in pressure volume loops were noticed but stress distribution was altered. Fiber distribution derived from diffusion tensor imaging should be considered for an accurate strain and stress analysis.

## INTRODUCTION

Cardiac tissue has a laminar structure with a preferred myocyte orientation in each layer. Given the syncytial nature of heart muscle, this preferred local direction is referred to as the fiber direction. An abundance of structural studies have documented changes to myocardial tissue architecture in aging and disease (1, 2). However, with the exception of rodents (3), few studies have conclusively linked these observations of diminished heart function to abnormalities of cardiac mechanics and/or electrophysiology arising from the disruption of fiber direction. This is due to the fact that cardiac electrophysiology and mechanics are tightly coupled and need to be studied simultaneously at multiple scales to be conclusive. Thus, in clinical practice, accurate electrophysiological characterization requires a minimally invasive biopsy (4), which is not compatible with the usual healthcare pathways.

With recent advances in computational tools for the multiscale modeling of cardiac electromechanics from the cell to organ level, it is now possible to determine more accurately the impact of structural changes within the myocardium on electromechanical cardiac function.

Such a framework needs precise experimental data as a baseline for the simulation. This relies mostly on multimodal clinical data with various levels of anatomical or functional detail, such as ultrasound, cine or tagged magnetic resonance image (MRI)-derived motion, computed tomography (CT)-derived segmentation, and late gadolinium enhancement (LGE) MRI-derived scar segmentation. However, patient-specific myocardial fiber orientation is lacking because of the combined respiratory and cardiac motion that challenge current acquisition methods. A broad panel of experimental methods is available to measure myocyte orientation ex vivo, which includes histology (5), μCT (6, 7), phase-contrast CT (8–11), and MRI (12, 13). In each case, the helix angle (HA) description is used to visualize the change in fiber orientation across the wall, which has been demonstrated in many mammalian species ranging from mice to human.

During the last decade, a growing number of studies have demonstrated that dynamic fiber orientation during cardiac contraction could be studied noninvasively in vivo by diffusion tensor cardiovascular magnetic resonance (DT-CMR) (14–16) or with three-dimensional (3-D) ultrafast ultrasound imaging (17), laying the foundation for quantification of structural and functional myocardial properties by in vivo assessment of fiber orientation. As a demonstration, recently, based on in vivo DT-CMR, the computation of the sheetlet angle in the cardiac reference frame was found to be a biomarker of dilated cardiomyopathy (DCM) (18). Nonetheless, the state-of-the-art solution remains too limited in spatial resolution (2 × 2 × 6 mm^3^) for creating detailed electromechanical models. Therefore, models must rely on the abundant ex vivo studies describing the organization of cardiomyocytes.

To date, the standard methodology for constructing patient-specific model geometries uses rule-based methods (RBMs), derived from canine hearts (19, 20). As underlined by Doste et al. (21), the fiber orientation distribution computed from RBMs mostly match histological data in the left ventricular (LV) free wall but larger discrepancies are noticeable, in particular, in the right ventricular outflow track (RVOT) or the interventricular septum (IVS) area. Indeed, some studies have mentioned the existence of a different fiber organization in the IVS. Although it is under debate, the existence of septal discontinuity has been observed in rabbit using X-ray phase-contrast tomography (11), in swine with histology (22) and DT-CMR (23), and in humans with gross pathology (24, 25) and ultrasound (26).

It is unclear how this deviation from the rule-based fiber orientation impacts electromechanical function. Vetter et al. (22) demonstrated a thin layer with fiber orientation significantly different from the underlying layers in swines. They showed evidence, from both computer simulations and optical mapping data, that this layer has a major impact on propagation and gives rise to unusual diamond and rectangular activation patterns.

The purpose of this study was to measure ex vivo species-diverse septal myocyte fiber orientation and assess its functional impact. In particular, it aims to detail the structure-function relationship in the interventricular septum by:

1. first performing a groupwise analysis of high-resolution ex vivo sheep hearts to create a biventricular template of diffusion tensor maps and identify the dual-layer organization of the fiber orientation. To expand the scope of the results, we analyzed the fiber orientation in five mammalian species (rat, canine, pig, sheep, and human) with high-resolution diffusion data. We also describe the potential interlocking of the layers with the conduction system;
2. investigating the electrophysiological effects of fiber orientation on local septal activation times. These analyses were based on the observed sheep experimental data;
3. finally, exploring the effects of the different septal fiber orientations on electromechanical output. The coupled 3-D–0-D fluid electromechanics framework introduced by Augustin et al. (27) is considered for the simulations on a 3-D image-based biventricular canine geometry.

## METHODOLOGY

As mentioned in introduction, analysis was performed in different species. The specific methodology used was as follows:

### Sample Preparation

1. Rat (*n* = 1, male, age: 6 mo), pig (*n* = 1, male, 4–6 wk), and sheep (*n* = 3, female, 2 yr) samples: The hearts were explanted via sternal thoracotomy under general anesthesia. The protocols were approved by the Animal Research Ethics Committee in accordance with the European rules for animal experimentation.
2. The dog sample (*n* = 1, male, adult) is an open-source dataset available on the John Hopkins website. We invite the reader to the following reference Helm et al. (2) for sample preparation.
3. Human sample: An ex vivo human donor heart was obtained from a 53-yr-old woman with acute coronary syndrome. The heart was obtained through the Human donor program “CADENCE” (providing access to heart samples from patients under cerebral death for scientific research purposes) approved by the French Agence de la Biomédecine. After written informed consent from the patient’s family was obtained, the heart was collected as a human biological sample for scientific research. The experiment was conducted in accordance with the declaration of Helsinki.

All hearts were fixed in a relaxed state with a solution containing formalin (10%) and gadolinium Dotarem (0.2%), by means of retrograde perfusion from the aorta. The sample preparation was performed as previously described by Magat et al. (28).

### Acquisition

All experiments were performed at 9.4 T with an open bore access of 30 cm (BioSpin MRI; Bruker, Ettlingen, Germany) using a gradient insert of 200-mm and 114-mm inner diameters for large and small samples, respectively. Images were scanned using a dedicated radiofrequency (RF) volume array coil with seven elements for pig, sheep, and human hearts. For the rat heart, a four-element surface coil as receiver coupled with a 72-mm diameter volume coil as transmitter was used. All MRI scans and experiment acquisitions were similar to those described previously by Magat et al. (28):

1. Diffusion-weighted images were carried out using a 3-D diffusion-weighted (DW) spin-echo sequence at an isotropic resolution of 600 μm^3^ and 200 μm^3^ for the large samples and rat heart, respectively. The DWI dataset consisted of six noncollinear diffusion encoding directions acquired with a *b* value of 1,000 s·mm^−2^.
2. A 3-D FLASH sequence was applied to get anatomical images of the whole heart volume, at an isotropic resolution of 150 μm^3^ for the pig, sheep, and human samples. The resulting volume is referred to as the anatomical image.

For the dog sample, note that the voxel resolution was anisotropic (312 × 312 × 800 μm^3^). We invite the reader to the following reference Helm et al. (2) for DW acquisition parameters.

### DW Images Preprocessing

2dseq files were converted in Nifti format with the dicomifier library (https://dicomifier.readthedocs.io/). A denoising was performed using the Marchenko-Pastur Principal Component Analysis (MP-PCA) algorithm (29). A B1 inhomogeneity field correction was applied with ITK-N4 (30) to reduce the intensity bias and signal drop-off in the apical and basal regions associated with the RF coil sensitivity profile.

### Diffusion Tensor Imaging Estimation

Diffusion tensor calculations were performed before any registration to avoid a difficult transformation of the diffusion encoding matrix. Diffusion tensor maps [eigen values: λ_1_, λ_2_, λ_3_, apparent diffusion coefficient (ADC), fractional anisotropy (FA), and color-coded FA (cFA), also known as red-green-blue colormap] were obtained with MRtrix3 software (https://www.mrtrix.org) (31). The first diffusion tensor eigenvector *v*_1_ corresponds to myofiber’s main orientation (13, 32). The second *v*_2_ and third *v*_3_ eigenvectors were associated with sheetlet in-plane and normal directions, respectively. The left ventricle was subdivided into regions using a polar coordinate system described previously (33), and finally, the helix (HA), transverse (TA), and sheetlet elevation (SE) and sheetlet azimutal (SA) angles were calculated for each sample. The myoarchitectural disarray index (MDI) introduced in an ex vivo cardiac diffusion study by Garcia‐Canadilla et al. (34) was computed using a region of interest (ROI) of 3 × 3 × 3 voxels (1.8 × 1.8 × 1.8 mm^3^). It describes the uniformity of the first diffusion tensor eigenvector *v*_1_ compared with its closest neighbors. Lower values indicating either a loss of myocyte organization, or that the orientation of the myocytes changes abruptly over a short distance.

### Template Creation and Diffusion Tensor Images Registration

A groupwise registration of diffusion tensor image was created with the three ex vivo sheep hearts. The steps performed to generate the diffusion tensor template are detailed as follows:

1. Prealignement: nondiffusion weighted images (b0) of each sample were registered manually with ITK-snap (http://www.itksnap.org/) (35) to align manually the long axis of the left ventricle to the *z*-axis of each volume and hence, obtain a similar alignment among hearts and ease the template construction.
2. Template creation: a groupwise registration was performed on the b0 images using the advanced normalization tools (ANTs) (36) to map the population to an average cardiac geometry in the so-called “template space” via the “antsMultivariateTemplateConstruction2.sh” procedure. The individual diffeomorphic transformations of the b0 images to the template were calculated with the symmetric diffeomorphic normalization (SyN) (37, 38).
3. Registration of diffusion tensor images: individual diffusion tensor images were warped to the template space. This process includes both registration of the voxel at the new location and reorientation of the tensor via the “antsRegistration” and “ReorientTensorImage” command of ANTs. Preservation of eigenvector orientations related to sample orientation from native space to template space were carefully checked during all the processing steps.
4. Averaging of diffusion tensor images: the transformed diffusion tensors were averaged via the “AverageTensorImages” (39) of ANTs to create the diffusion tensor template. Finally, the diffusion tensor maps and angle maps were computed as described in the previous section.

The other samples were also individually aligned and registered to the template using Affine transformation for visualization purpose.

### Tractography Processing

Tractography was used for visualization of global myofiber arrangement. All the tractography steps described in this section were performed with MRtrix3 (40). Streamlines were generated with the FACT algorithm (41). They can be computed in the native space using the primary eigenvector of the individual diffusion tensors or in the template space using the primary eigenvector of the average diffusion tensor. The average diffusion tensor is depicting an averaged myofiber organization across samples. We defined the tractography parameters as an FA-stopping threshold of 0.1, a maximum angle between steps of 60°, a step size of 0.05 mm, a maximum length of 40 mm, and a minimum length value of 1 mm.

## COMPUTATIONAL MODELS

### Sheep Septal Geometry

**Figure 1.**
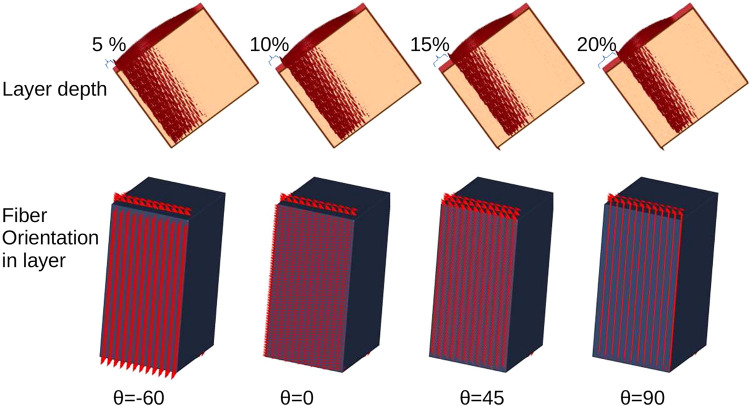
*Top row*: depiction of the layer constructed in each scenario, ranging from 5% to 20% of the total length in the transmural direction. *Bottom row*: angle configuration in the layers, cases considered in our study: 45°, 0°, −45°, −60°, and −90°. For brevity of exposition, only four configurations are shown.

A 2.1 cm × 1.8 cm × 3.5 cm wedge, discretized at 300 μm along each dimension, yielding 500,000 nodes and 2.5 million tetrahedra, was constructed to represent the septum of a sheep heart (see Fig. 1).

**Figure 2.**
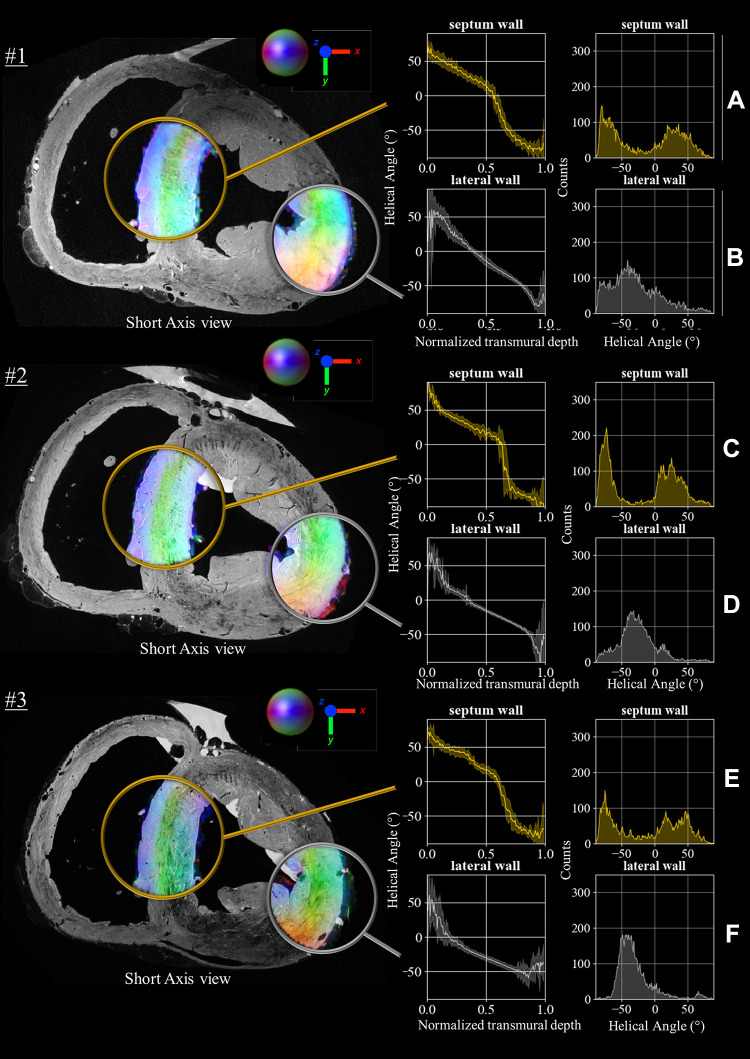
Anatomical images and diffusion tensors metrics in lateral and septal wall for *n* = 3 sheep samples. Anatomical images (at 150 µm isotropic voxel size) are overlaid with color-coded fractional anisotropy (cFA) maps (at 600 µm isotropic voxel size), that encodes the *x-y-z* coordinates of the principal eigenvector *v*_1_ derived from the diffusion-weighted (DW) images. The white circle highlights typical fiber orientation described by the rule-based fiber models in the mid-lateral part of the left ventricle. The yellow circles highlight both inferior-superior (IS) orientation of fiber in the epicardial part of the septum and typical anterior-posterior (AP) orientation of the fibers in the endocardial part. For each sample, the transmural variation in helical angle and the histograms of helix angle across the two regions are indicated in the right panel for septal (*A*, *C*, and *E*) and lateral wall (*B*, *D*, and *F*) in mid-high areas.

Based on the data (*n* = 3 sheep hearts), we computed the thickness of both left and right ventricular layers, as well as the variation in angle orientation across the wall, after which we computed the average of them. We then mapped the averaged angle measurements (inside the colored circles covering the septum shown in Fig. 2) of the three hearts to our wedge using interpolation by inverse distance weighting.

### Dog Heart

A canine biventricular geometry obtained at end diastolic phase from a cardiac magnetic resonance image (MRI) of a 20.6-kg mongrel dog (27) was used. The finite element discretization was generated using CGAL (http://www.cgal.org/) and smoothed using meshtool (42). The discretized geometry consisted of 111,234 points and 557,316 tetrahedral elements. To provide closed cavities for the biventricular geometry, the valves were replaced by a one tetrahedral element thick layer. The fibers were constructed using a rule-based algorithm (43) with fiber angles of 60° and −60° and sheet angles of −65° and 25° on the endocardial and epicardial surfaces, respectively.

**Figure 3.**
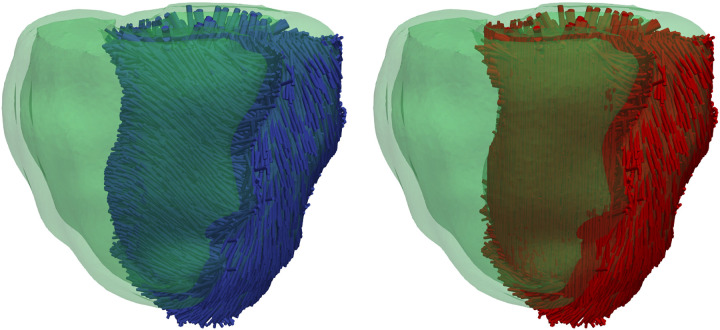
*Left*: usual rule-based fibers configuration used as control in our studies. *Right*: geometry with a modified region where the fibers are oriented from base to apex, and outside this region they follow the usual rule-based configuration.

To explore whether there are any electromechanical changes from the experimentally observed fibers that did not follow the rule-based algorithm, we generated three different dog geometries that had a layer of variable thickness in which the fibers pointed downward. We named these three geometries layer20, layer30, and layer40. The respective number accounts the thickness (in percentage) of the septal layer in which the fibers pointed in the apicobasal direction. We used the previously described Universal ventricular coordinates (UVCs) (43) of our original geometry to construct these three auxiliary geometries. Concretely, for the layer40 geometry, we modified the fiber direction of the mesh where the transmural coordinate (ρ) in the UVCs was greater than 0.6 in the left ventricle, the height coordinate was in the range 0.35 < *z* < 0.95, and the circumferential coordinate (φ) was in the range 0.3 < φ < 0.7. For the two remaining cases, layer30 and layer20, we used the same values for the coordinates *z* and φ as in the layer40 case, and we chose the transmural coordinate (ρ) to be greater than 0.7 and 0.8, respectively. Figure 3, *left*, shows the dog biventricular mesh with the rule-based fibers that served as a control for our studies, whereas Fig. 3, *right*, shows one of the auxiliary geometries (layer40) where the fibers were oriented in the apicobasal direction for the layer defined on it.

### Mechanics

The ventricular walls were modeled as an orthotropic medium (44), written as

(*1*) $$\text{Ψ(E) =}\frac{C}{2}\left(e^{Q}-1\right)+\frac{k}{2}{ln}^{2}\left(J\right),$$

$$Q\;=b_{ff}E_{ff}^{2}+2b_{fs}E_{fs}^{2}+2b_{fn}E_{fn}^{2}+b_{ss}E_{ss}^{2}+b_{nn}E_{nn}^{2}+2b_{sn}E_{sn}^{2},$$

where Ψ is the strain energy function; *J* is the Jacobian of the deformation gradient *F*; $E_{ff}$, $E_{ss}$, and $E_{nn}$ are the axial strains along the fiber, sheet, and normal directions, whereas $E_{fs}$, $E_{fn}$, and $E_{sn}$ are the rotational strains in the fiber-sheet, fiber-normal, and sheet-normal planes, respectively, and *C*, $b_{ff}$, $b_{fs}$, $b_{fn}$, $b_{ss}$, $b_{sn},$ and $b_{nn}$ are the passive material parameters and *k* is the incompressibility factor.

The base of the heart was modeled by a stiffer neo-Hookean material, to account for the rigidity of the tissue, with the addition of the incompressibility penalty term, as shown in *Eq. 2*

(*2*) $$\text{Ψ(E) =}c\left(I_{1}-3\right)+\frac{k}{2}{ln}^{2}\left(J\right)$$

where *I*_1_ is the first invariant of the Cauchy–Green strain tensor and *c* is the material parameter.

### Electrophysiology

Electrical activity in the sheep septum was modeled with the monodomain formulation. The ten Tusscher model (45) was used with modifications in *G*_kr_ and *G*_Ks_ to replicate action potential duration in sheep hearts. These modifications are detailed in *Effect of Different Fiber Orientations on Electrical Activation*. The time step used was 20 μs with a temporal output of 1.0 ms for all simulations. The total duration of each simulation was 250 ms.

For the second part of our studies, in the canine biventricular mesh, because the geometry considered was too coarse for the use of the monodomain equation to simulate the electrical propagation in the tissue, we considered the hybrid reaction-eikonal model R-E^+^ (46). This model combines the standard monodomain equation with an eikonal term for the propagation velocity, and is written as

(*3*) $$C_{m}\frac{\partial V_{m}}{\partial t}=I_{foot}+\nabla \cdot \sigma_{i}\nabla V_{m}-I_{ion}$$

where *C*_m_ is the membrane capacitance per unit area, *V*_m_ is the transmembrane voltage, σ_i_ is the intracellular conductivity tensor, *I*_ion_ is the ionic current, and *I*_foot_ is a stimulus current, as detailed by Neic et al. (46). This model is suitable for lower resolution geometries such as the one considered here.

Also at a cellular level, the ten Tusscher ionic model is considered for the canine ventricular myocytes.

### Electromechanical Coupling

The active tension model developed by Land et al. (47) simulates the generated contraction force of ventricular myocytes. This model consists of six ordinary differential equations (ODEs) and incorporates a length- and velocity-dependence on the generated tension and the calcium sensitivity. The generated tension *T*_a_ is written as

(*4*) $$T_{a}\left(\lambda ,\dot{\lambda }\right)=h\left(\lambda \right)\frac{T_{ref}}{r_{s}}\left(\left(\zeta_{s}+1\right)S+\zeta_{w}W\right)$$

where λ is the stretch ratio, λ̇ is the rate of change of the stretch ratio, *h*(λ) is a sensitivity function of the stretch, *T*_ref_ is the maximal active tension at resting length, *r*_s_ is the steady-state duty ratio of the presented three-state crossbridge model, and ζ_s_ and ζ_w_ are the distortions at the pre-powerstroke state *W* and force generating post-powerstroke state *S*. The calcium sensitivity to the stretch affects the two states *W* and *S* in the induced tension model and introduces a one-way coupling with the ten Tusscher model, which provides the intracellular calcium.

### Model Parameterization

The computational model was numerically solved using the finite element method via the Cardiac Arrhythmia Research Package (CARPentry) software (48, 49), built upon extensions of the openCARP EP framework (http://www.opencarp.org) (50). The parameters for the passive properties of the myocardium *b*_ff_, *b*_fs_, *b*_fn_, *b*_ss_, *b*_sn_, and *b*_nn_ match those used by Usyk et al. (44), since the model was tuned to simulate a canine heart. We tuned the parameter *C* = 1.6 to match the physiological range for ventricular end-diastolic volumes. A spring stiffness *c* of 1 MPa was considered for the base of the heart. In both materials, the incompressibility factor *k* was set to 650 kPa.

Eikonal parameters were tuned to obtain a QRS duration that laid in the physiological range for canines. In particular, the velocities were tuned as (*v*_f_, vs. *v*_n_) = (0.36, 0.24, 0.12) in m/s and the conductivities as (*g*_f_, *g*_s_, *g*_n_) = (0.4, 0.5, 0.5) in S/m.

The base of the heart was considered nonconducting; thus, the conductivities were set to zero. We tuned the active tension model to obtain a peak pressure within the physiological range, and to match the pressure-volume (PV) loop profiles of the left ventricle that appear in the study by Maughan et al. (51). We used the values given by Land et al. (47) with the exception of *T*_ref_ = 100 and [Ca]_50_ = 0.9.

Since tension is distributed in both the fiber and the transverse direction and can reach up to 50% in the latter relative to the former (52, 53), we consider that 40% of the tension acts transversely. The parameters of the 0-D CircAdapt model of the cardiovascular system, which is coupled to the 3-D electromechanical model of the ventricles according to Augustin et al. (27), are tuned to match the end-diastolic volume of the geometry and to obtain results that lie within the physiological ranges. Regarding the pericardial boundary conditions, stiffer springs were considered near the apex of the geometry (54). The stiffness varied from the apex to the base by 0.001 kPa/μm. The cardiac cycle length was 600 ms, corresponding to 100 beats/min. The entire endocardial surfaces of both ventricles were stimulated to mimic Purkinje activation.

## RESULTS

### Measured Fibers in Sheep Data

Figure 2, *left*, shows in short axis orientation the experimental anatomical images acquired using the 3-D FLASH sequence at 150 μm^3^ on the three ex vivo samples. The images are warped to match the template, so all images are placed in the same spatial reference for further processing and comparison of metrics. The cFA maps that encode the *x-y-z* coordinates of the principal eigenvector (*v*_1_) derived from DW spin-echo sequence are superimposed in the IVS and the free (or lateral) wall. Smooth transitions of fiber orientation are visible (a color gradient of purple to green) in the lateral wall while a discontinuity in the septum is highlighted by the green color that depicts anterior-posterior (AP) orientation of the fibers and the blue color that depict inferior-superior (IS) orientation of fibers.

In Fig. 2, *right*, the boxes show the transmural variation of the helical angle from 3-D ROI located in the basal area in the two areas (IVS and lateral). The number of voxels in the septum and lateral ROI for each sample is as follows: *1*) 9,107, *2*) 7,549, and *3*) 7,485 and *1*) 12,111, *2*) 12,062, and *3*) 9,576, respectively.

In all samples, a typical fiber orientation described by the rule-based fiber models is visible in the lateral wall, while we notice a sudden drop of the helix angle close to two-thirds of the wall, suggesting the presence of two distinct layers of fibers in the septum. A summary of the transmural distance where the discontinuity occurs is presented in Table 1. The distribution of helix angle along the transmurality also reported the presence of two populations in the septal area while one population is visible in the free wall.

**Table 1.** Transmural distance of the discontinuity and fiber orientation in the LV and RV endocardial surface

Animal	LV Layer, %	RV Layer, %	Orientation LV Endo	Orientation RV Endo
*Sheep 1*	61.9	38.1	60.7	−89.4
*Sheep 2*	58.3	41.7	51.6	−81.6
*Sheep 3*	62.1	37.9	58.4	−81.3
Average	60.8	39.2	57	−84

Data show 3 specimens considered in our study and an average of them. LV, left ventricular; RV, right ventricular, Endo, endocardium.

Supplemental Figs. S1 and S2 (all Supplemental Figures are available at https://doi.org/10.5281/zenodo.6469798) show for each sample the HE and SA angle measurements (solid lines) along with the standard deviation (shaded areas) as function of wall transmurality in septal, lateral, anterior, and posterior wall. For each, the sample was divided in four subregions (apical, mid-low, mid-high, and base) along the *z*-axis (or long axis). The sudden drop in HA and rise in SA is more pronounced closed to the base than to the apex. Supplemental Fig. S3 shows for the large mammalian samples (pig; sheep 1, 2, and 3; and Human), the anatomical images, the cFA maps, the helix angle maps, and the myocardial disarray index (MDI) maps in the long axis orientation in the IVS. The delineation of the dual layer is well depicted using both the MDI and the cFA maps. The metrics indicate that the layer is present from base to apex in animal samples (black arrow) whereas less sharp in the pig sample. In the human sample, the metrics indicate that the layer fade close at the level of the moderator band (pink arrow). Lower values are also visible in the mid low area because of the presence of trabeculation in the right ventricle (purple arrow).

**Figure 4.**
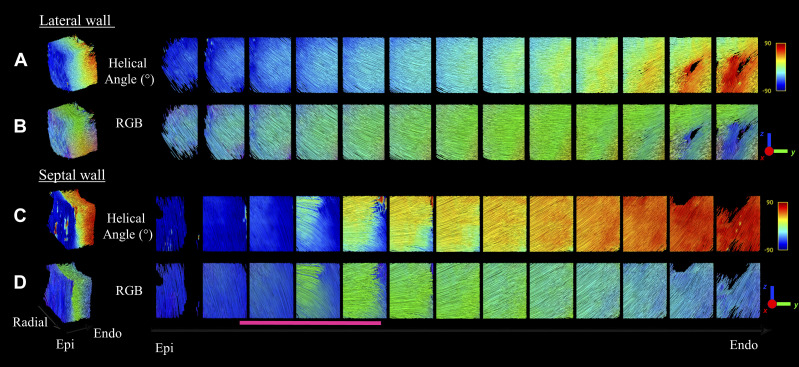
Local tractography in septal and lateral wall using the diffusion tensor template. A box in the left ventricle is being viewed from the epicardium to the endocardium in the septal (*A* and *B*) and in the lateral wall (*C* and *D*). Streamlines are color-coded using the helix angle (*A* and *C*) and the color-coded fractional anisotropy (cFA) maps (*B* and *D*). The cFA maps color-code corresponds to the direction in the anatomical frame: green, anterior-posterior; blue, inferior-superior; and red, left-right. In the lateral wall, the streamline smoothly rotates from the subendocardial to subepicardium. In the septum wall, a discontinuity is visible close to the epicardial part (pink line) where streamlines rotate between 60° and 90° from antero-posterior direction to inferior-superior direction.

A zoomed view of streamlines describing the rotation of the fiber arrangement of the diffusion tensor template is shown in Fig. 4 in both the septal and lateral area. The streamlines are color-coded using both the helix angle and the cFA maps. In the lateral area, myofiber arrangement smoothly rotates from the subendocardium to subepicardium. In the IVS, a clear gap or discontinuity is visible close the epicardium (red line) where streamlines rotate from almost 90° from the antero-posterior direction to the inferior-superior direction.

### Visualization of Myofiber Orientation in the Interventricular Septum in Five Mammalian Species

**Figure 5.**
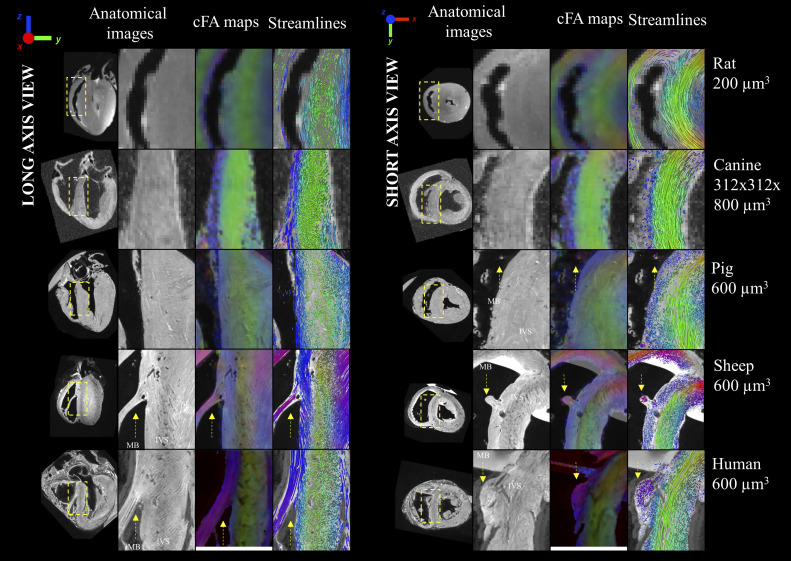
Anatomical images and diffusion tensors metrics in the intraventricular septum for five mammalian species (rat, canine, pig, sheep, and human). For each species, a large view, a zoom view of the anatomical, the color-coded fractional anisotropy (cFA) maps, and the streamlines are shown in long-axis (*right*) and short-axis (*left*) views. For each sample, a predominant fiber direction (in green color) displays the well-known circular arrangement of fiber in the left ventricle (LV). A second layer of fiber with an up-down orientation (in blue color) is visible for all species except for the rat. For the sheep and human sample, the visualization of the streamlines shows a clear continuity between the fiber orientation in the moderator band (indicated by yellow arrows) and the interventricular septum (IVS).

Anatomical images, cFA maps, and streamlines are shown in long-axis (Fig. 5, *left*) and short-axis (Fig. 5, *right*) views for five different species (rat, canine, pig, sheep, and human). For each sample, a predominant fiber orientation (in green color) displays the well-known circular arrangement of fiber in the LV. The presence of a layer with fiber orientation in the up-down direction suggests the presence of two distinct layers of fibers all along the septum in all large mammalian species.

At 0.150-mm resolution, the moderator band (white arrows) can be detected in the anatomical images for the pig, sheep, and human hearts. In the sheep and human sample, the diameter of the moderator band (close to the 2 and 4 mm, respectively) was sufficient, using the diffusion weighted images, to map the fibers connecting to the interventricular septum. The visualization of the streamlines shows a clear continuity between the fiber orientation in the moderator band and the layer in the right part of the IVS. In the pig sample, the diameter of the moderator band (less than 1 mm) was insufficient with regard to the spatial resolution of the acquisition scheme.

### Effect of Different Fiber Orientations on Electrical Activation

#### Sheep septum.

From the literature (55, 56), we know that conduction velocity in sheep ventricles in the longitudinal direction ranges from 50 to 100 and 20 to 40 cm/s for the transmural direction. Based on these, we chose for our studies a conduction velocity of 75 and 30 cm/s for the longitudinal and transversal directions, respectively.

We tuned the parameters in each dimension to obtain the optimal values for modeling these aforementioned velocities, and we obtain, for the longitudinal direction, *g*_i_ = 0.2390 and *g*_e_ = 0.8585. For the transverse direction, we have *g*_i_ = 0.0382 and *g*_e_ = 0.1374, respectively.

Also, from the literature (55, 56), we know that the action potential duration (APD) in the left ventricle is roughly around 300 ms. We modified the ten Tusscher model to get an APD of around 300-ms duration by decreasing both *G*_Kr_ and *G*_Ks_ by 20%.

To get insight into the effect of fiber orientation on septal propagation, we constructed an idealized wedge representing the septum. We created a layer representing 5%, 10%, 15%, and 20% of the total thickness of the septum starting from the right ventricular endocardial surface and going transmurally. For each of these cases, the fiber orientation in said created layer was modified. We used five different configurations of fibers: −90°, −45°, 0°, 45°, and 90°. Outside this layer, we used the averaged data of our segmented hearts, and we set the fibers by means of Laplace–Dirichlet rule-based algorithm (20). Figure 1 shows a scheme of the study.

For each scenario, we initiated propagation in the center of the right ventricle endocardium in the wedge. The strength of the transmembrane stimulus delivered was 100 μA/cm^2^ delivered at the center of the endocardial surface.

**Figure 6.**
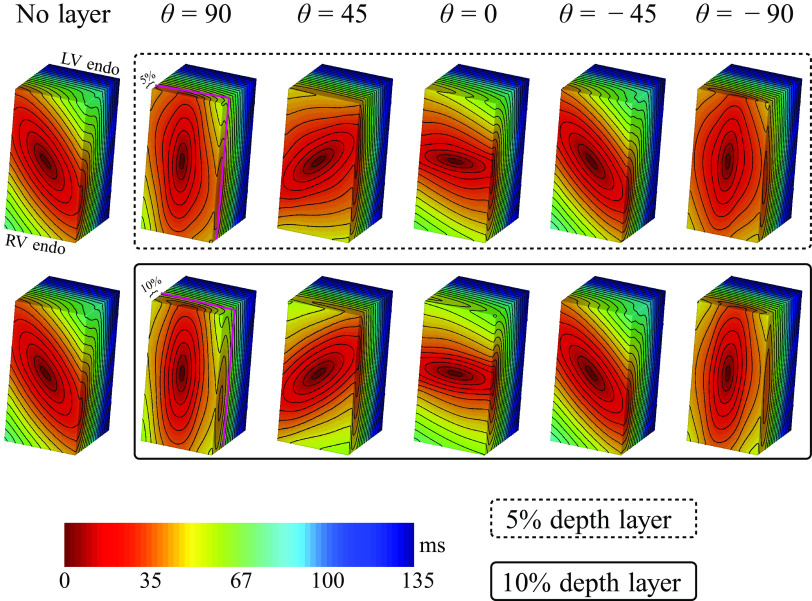
Activation time maps for the different cases considered in our study. Left column shows the usual case with no layer defined in it to serve as control. Inside the dashed rectangle, we show the case where the depth of the layer accounts for 5% of the total transmural depth and five different angle configurations inside the layer, θ = 90°, 45°, 0°, −45°, and −90°, respectively. The pink line indicates the border of the layer. Inside the solid line rectangle, an analogous is shown for the case when the depth of the layer accounts for 10% of the total transmural depth and the same five angle configurations. Again, the pink line delimits the layer. In all cases, activation was initiated by pacing at the center of the concentric ellipses.

In all these cases, and more easily distinguished for 5% and 10% total thickness of the layers, a diamond shape wave front was observed because of the transition of the wave propagating front between the two layers, in accordance with the work reported by Vetter et al. (22) for other species (swine hearts), the only difference being the time at which these fronts appeared. Examples of propagation for every combination of layer depth and fiber orientation are shown in Fig. 6 where we show isochrone maps of the respective scenarios. For the cases where we have deeper layers (15% and 20%), it is possible to observe the diamond shape propagating fronts, but as it takes more time to reach that transition zone, these layers would need to be immersed in an even larger wedge for us to observe the fronts as in the cases with 5%- and 10%-layer depths.

### Effect of Different Fiber Orientation on Mechanical Output

#### Dog heart.

**Figure 7.**
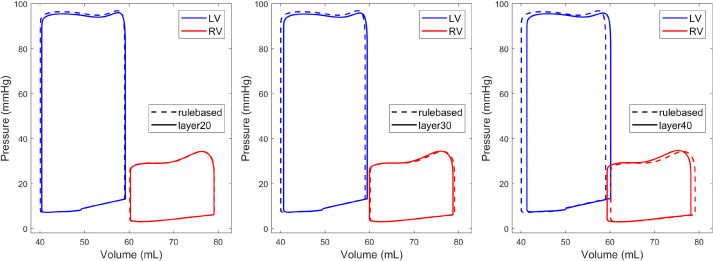
The pressure-volume loops of the left and right ventricles (LV and RV, respectively) for a simulated heartbeat with a layer of 20% (layer20, *left*), 30% (layer30, *middle*), and 40% (layer40, *right*) of fibers pointing in the apicobasal direction in the septum, compared against the rule-based fiber model (dashed line). Minimal differences are observed for all models regardless of the use of a layer of apicobasal pointing fibers in the septum.

We observed minor differences in the pressure-volume loops, as shown in Fig. 7. The stroke volume of the LV increased as the thickness of the layer with the fibers pointing in the apicobasal direction increased, whereas the one of the RV followed the opposite trend. The maximum difference in the stroke volume of the ventricles was 1 mL, which was observed for a layer of 40% thickness.

**Figure 8.**
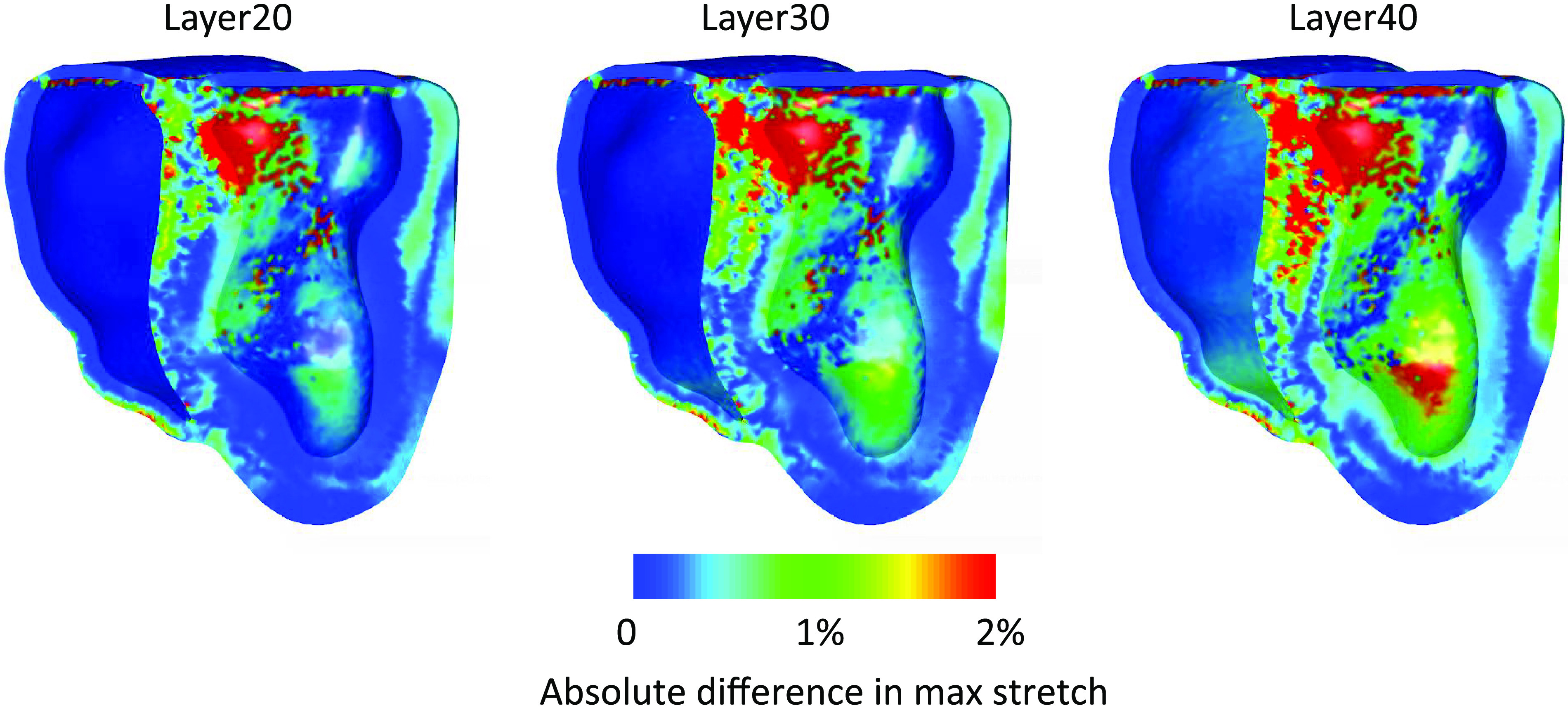
The absolute difference of the maximum stretch over a heartbeat of each model that contains a layer of 20% (Layer20), 30% (Layer30), and 40% (Layer40) of fibers in the septum pointing in the apicobasal direction against the model with the rule-based fibers. For a thin layer of 20%, 2%, or higher differences in stretch appear in the septum, while minor differences are present in the left ventricle (LV) and right ventricle (RV) walls. As the layer increases to 40%, significant differences in the stretch appear in a wider area of the septum and near the apex of the LV. In addition, areas of about 1% differences are present in both ventricles in the case of 40% layer thickness.

**Figure 9.**
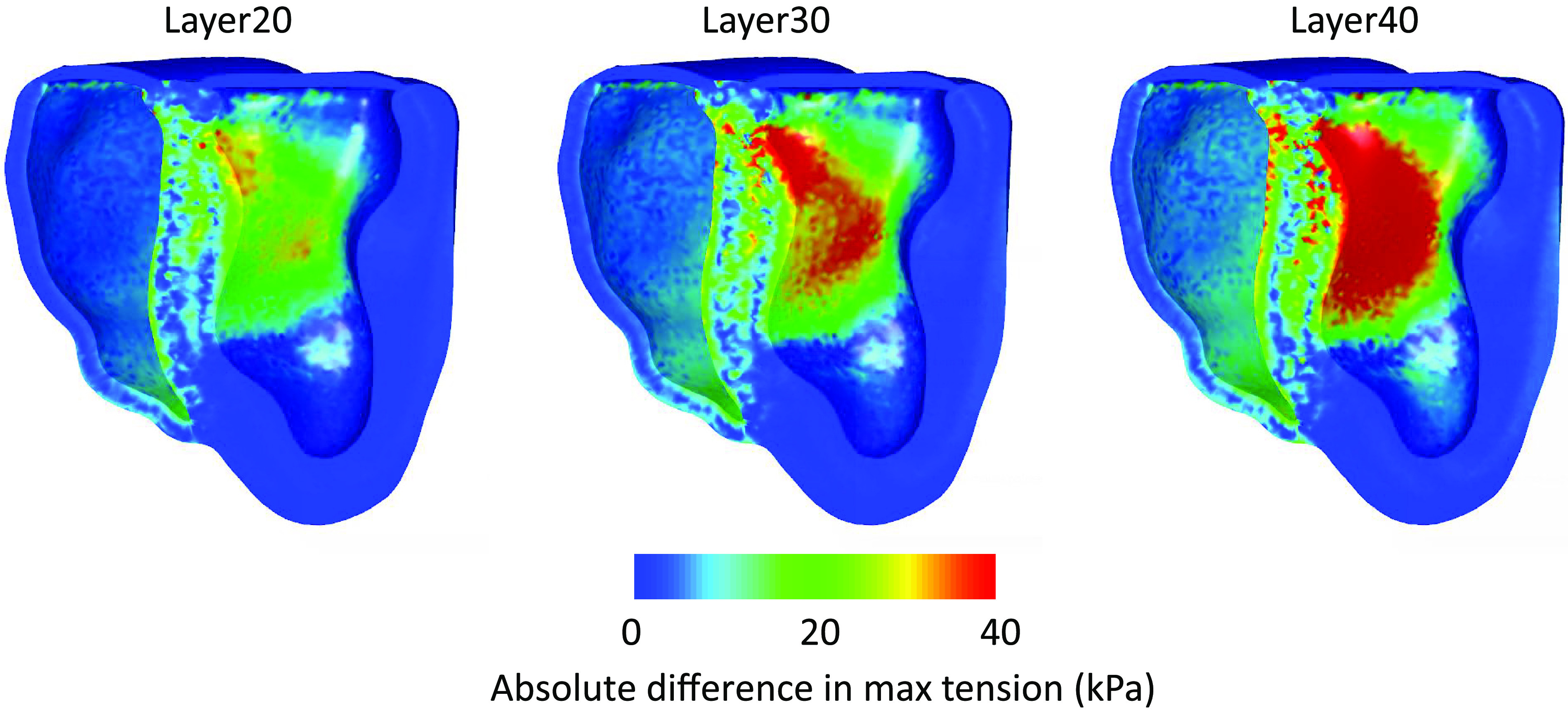
The absolute difference of the maximum tension over a heartbeat of each model that contains a layer of 20% (Layer20), 30% (Layer30), and 40% (Layer40) of fibers in the septum pointing in the apicobasal direction against the model with the rule-based fibers. For a thin layer of 20%, the largest difference in the tension appears in the septum; however, the tension difference does not exceed the 40 kPa. As the layer increases to 40%, significant differences in the tension appear in a wider area of the septum, with differences reaching 40 kPa or higher. Differences of nearly 20 kPa can also be observed in a larger area of the right ventricle (RV) for 40% layer thickness model. Note that the maximum tension differences lie near the endocardium, and they become smaller within the intraventricular septum wall.

We next compared the maximum stretch and induced tension over a heartbeat of the models with the different layer where the fibers are oriented in the apicobasal direction against the simulation with the rule-based fibers. Figures 8 and 9 present the absolute difference of the quantities, where differences near zero indicate that the quantities are similar to the baseline model (the rule-based fiber model). We observe that the difference in the maximum stretch over a heartbeat is more localized to the septum for a thin layer, while it expands on other regions of the heart, especially near the apex as the layer becomes thicker. On the other hand, the difference in the maximum induced tension is mostly localized at the septum and increases as the apicobasal pointing fiber layer thickens.

**Figure 10.**
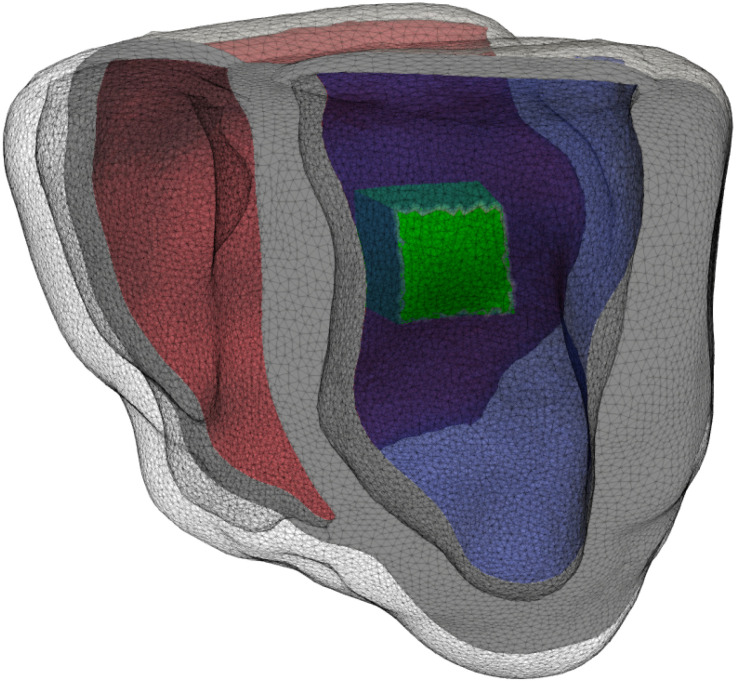
The area of the septum (in green) considered for the strain-stress analysis, ranging from the endocardial surface of the right ventricle (in red) to the one of the left ventricle (in blue).

To explore further the differences that appear in the maximum tension and stretch over a heartbeat, we considered an area in the septum that includes part of the area where the maximal differences occur, and we performed an analysis of the stresses and strains in the principal directions (f, fiber; s, sheet; and n, normal). A visual representation of the patch considered in the septum appears in Fig. 10 in green.

**Figure 11.**
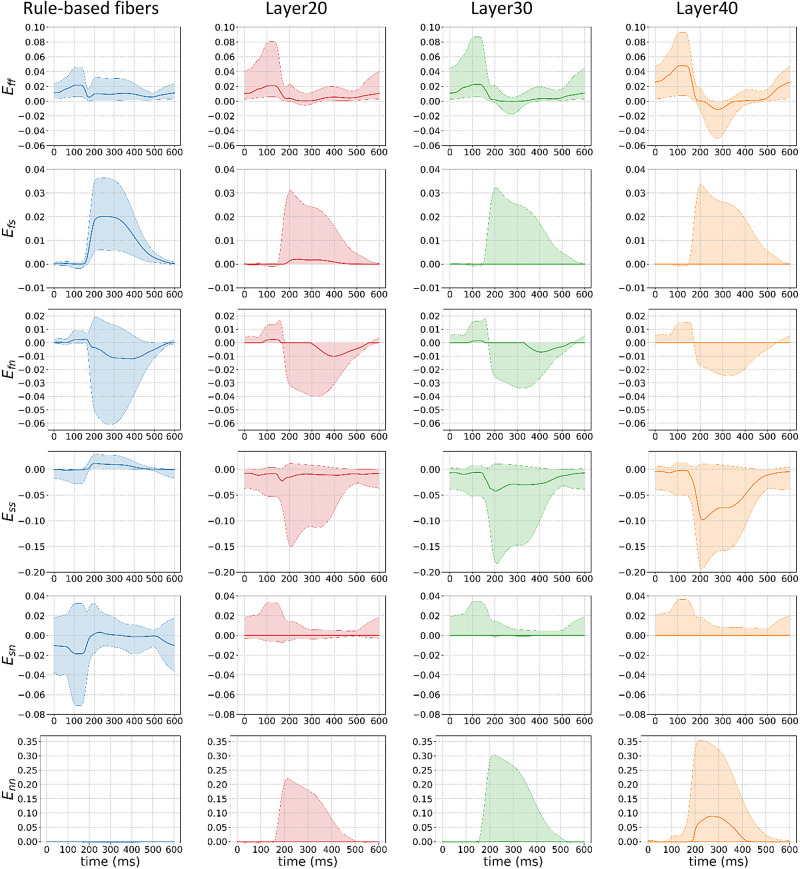
Strain analysis in the principal directions (f, fiber; s, sheet; and n, normal) for the axial (ff, ss, and nn) and rotational (fs, fn, and sn) strains for the four models (rule-based fibers in blue), and the models with 20% (Layer20), 30% (Layer30), and 40% (Layer40) of fibers in the interventricular septum (IVS) pointing in the apicobasal direction. The solid line is the average whereas the shaded area is the interquartile range. We observe two different trends: an increase in the magnitude of the axial strains in the fiber layer models and a decrease in the one of the rotational strains, when compared with the rule-based fiber model.

**Figure 12.**
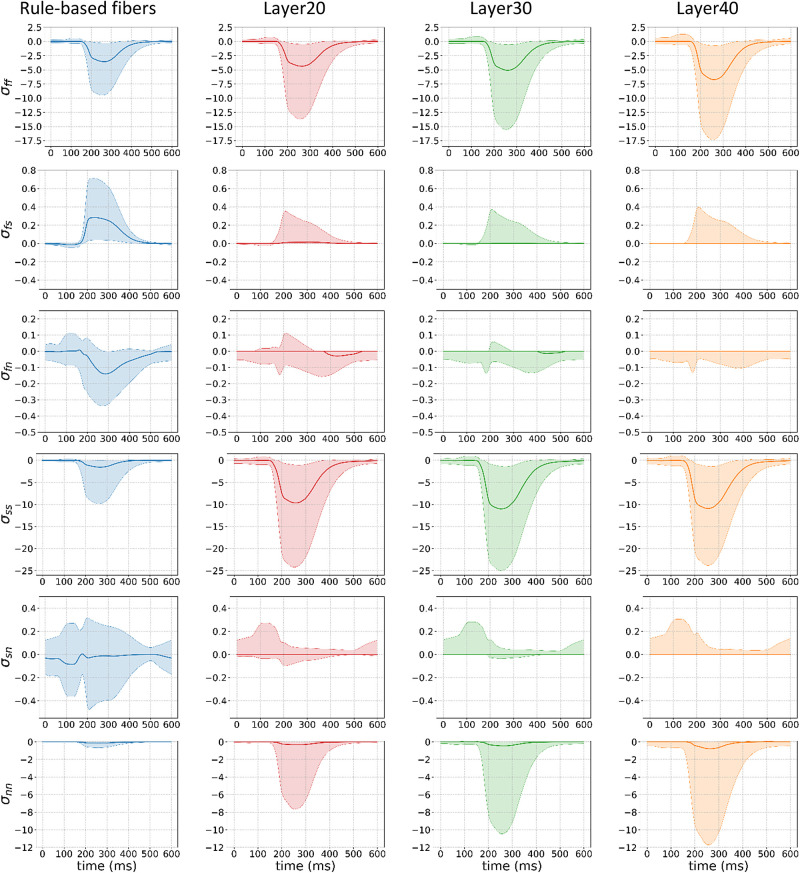
Stress analysis in the principal directions (f, fiber; s, sheet; and n, normal) for the axial (ff, ss, and nn) and rotational (fs, fn, and sn) stresses for the four models (rule-based fibers in blue, and the models with 20% (Layer20), 30% (Layer30), and 40% (Layer40) of fibers in the interventricular septum (IVS) pointing in the apicobasal direction. The solid line is the average whereas the shaded area is the interquartile range. The same trends as in the strain analysis are observed for the stress distributions.

We compared the stresses (in kPa) and strains in the principal directions for all four fiber configurations along a cardiac cycle. In particular, Figs. 11 and 12 show the interquartile range (shaded area) and the average (thick line) of the corresponding strains and stresses. This metric was chosen to avoid extreme values (outliers) that could be produced from the numerical simulations.

We observed that the distribution of the strain and stress is different even with a layer of fibers pointing in the apicobasal direction as thin as 20%, when compared with the rule-based fiber model. In particular, the average rotational stresses and strains are close to zero for the entire cardiac cycle and their interquartile distribution becomes smaller as the apicobasal pointing fiber layer thickens. On the contrary, the rotational stresses and strains play a major role during the ejection phase in the rule-based fiber model.

On the other hand, the presence of the layer increases the magnitude of the average axial stresses and strains and the interquartile range of their corresponding distributions, when compared with the rule-based fiber model. Although this effect is evident in all axial directions, it is particularly dominant in the sheet and normal directions of the fibers, where the interquartile range of the strains increases by an order of magnitude, following the increase in the corresponding stresses. This effect becomes more evident as the thickness of the layer increases.

## DISCUSSION

### New Anatomical Details

A small number of studies in the early 2000s reported that the architectural arrangement of cardiomyocytes in the IVS is one of two distinct layers in several species, including rabbit (11), pig (22), and human (26). With the exception of Hayabuchi et al. (57), Doste et al. (21), Agger et al. (58), and Ferreira et al. (59), these studies have not been subsequently replicated. Although advanced imaging tools are now available, the description of the IVS and the existence of a septal discontinuity remains under debate: some studies remove the right ventricular portion of the septum from their analysis (15, 60) whereas others refute the idea of bilayered ventricular septum (58). Finally, if it exists, the dual identity of the septum is not taken in account, neither in anatomical description nor cardiac modeling of the ventricle. In this work, we clarified septal architecture using new, high resolution, experimental data, and explored the functional consequences.

First, we highlight a discontinuity in the transmural variation of the helical angle in ex vivo sheep samples (*n* = 3) and we quantified the location of the discrepancy in the septal wall (40%–60%). The helix angle distribution found agreed with previous work (61) (Fig. 1 in ovine model with *n* = 11). To extend the scope of the result, we observed for the first time in four mammalian species (dog, pig, sheep, and human) a similar fiber orientation in the septum. Although cFA maps and streamlines depict a similar pattern in large mammalian samples, the existence of a bilayered fiber orientation was not identified in the rat breed. The limited resolution of the acquisition (200 μm^3^) could explain this result; indeed, a similar transmural variation of the helix angle was found in a murine study (38) (*n* = 5) at a voxel resolution of 43 μm^3^ where an inflection point between mid- and epi-mid-transumral depth of the septum is noticeable (Fig. 6). In addition, a morphogenesis study (62) of the septum in rodent (transgenic mouse lines) demonstrated the existence of right and left ventricular myocardial cell populations during formation of the IVS.

Debates about the existence of a bilayered fiber orientation and associated echogenic septal area have also been reported in the literature. Whereas Hayabuchi et al. (57) suggests that the bright line observed by in vivo ultrasound is a demarcation line between the ascending and descending segments of the heart, Agger et al. (58) demonstrate that the echogenic zone is due to the perpendicular orientation of the myocites with respect to the angle of incidence of the beam. As a result, this effect is visible in the midwall area but is not limited to the septum. Here, ex vivo MRI data in the ovine breed indicate a change in the diffusion tensor metrics and the associated putative myofiber/sheetlet arrangements at one-third of the septum wall. Thus, we noticed a change in helix angle and sheet azimuthal angle and a decrease in FA. Although the location of this discontinuity is in the midwall area, the spatial resolution of the acquisition is sufficient to accurately estimate this location. To investigate whether the discontinuity in fiber orientation changes from base to apex, different metrics all computed from the first diffusion tensor eigenvector *v*_1_ are presented in Supplemental Fig. S3. The discontinuity is present in the basal area in the human sample and fades after the moderator band, whereas in other samples, it persists until the lower right ventricular attachment point. The interpretation of the quantitative metrics remains subject to discussion. The presence of the layer is depicted by the helix angle maps but is more subtle to distinguish at the apex. In comparison, both MDI and the cFA maps clearly depict a discontinuity in fiber orientation. The decrease in FA at the discontinuity is likely due to a partial volume effect with the presence of multiple fiber orientations within a voxel, but a slightly different myofiber architecture could also be considered. For example, the cellular organization of the discontinuity cannot be assessed by MRI, but we can speculate that this area might be more sensitive to structural remodeling. The change in fiber orientation and cleavage plane could increase intracellular spaces and promote either fibrosis or fat infiltration. Myocardial damage has been reported in this specific area by LGE (63) and IDEAL (64) MRI.

### Purkinje Network Implications

The superficial RV septal endocardial muscle tracts that we report on here are oriented in the same direction of the RV Purkinje system fascicle. This is further evidenced by the continuity of the tract with the moderator band, which has a Purkinje fiber embedded within it to activate the papillary muscle on the RV free wall. Since Purkinje activation is vital, the layer may be important for the Purkinje fiber growth since the conduction system tends not to grow across fiber direction but along it, much as it follows trabeculation in the LV. The Purkinje system fibers running on the RV septum do not form electrical junctions with the myocardium above the moderator band, with activation of the RV septum resulting from transeptal propagation initiated by the left ventricular septal fascicle. Below the septal attachment of the moderator band, the fascicle forms junctions to activate the apical area.

### Effect on Electromechanical Function

Typically, state-of-the-art computational models consider rule-based fibers for the electromechanical simulations of the heart. The dual layer effect produced a diamond activation pattern as previously noted in swine (22). Although the effect of the fiber orientation on a canine biventricular geometry was minimal on the resulting pressure and volume of the heart, the strain and stress distribution were considerably different. In particular, without neglecting the anatomical variability between species and the functional implication, our results indicate that the magnitude of the axial strains and stresses is underestimated whereas the rotational is overestimated. Altered local stresses and strains could lead to growth remodeling that may have important longer-term consequences. Thus, a fiber distribution derived from diffusion tensor imaging should be considered for an accurate strain and stress analysis.

### Imaging Methodology Advancement

The current study relies on a dedicated sample preparation and acquisition method. First, all samples were perfused and fixed with the same fixation protocol. Hearts were flushed with a cardioplegic solution that relaxed cardiac muscle before formalin fixation. The fixation solution included a gadolinium-based contrast agent to increase the signal-to-noise ratio. The acquisition methods were specifically developed for large-sized samples and relied on a high-field magnet at 9.4 T with an open bore access of 30 cm combined with a unique RF volume array coil of seven elements with an inner diameter of 165 mm and a dedicated preparation module for homogeneous B1 field and excitation (28). Such a combination allows anatomical and diffusion-weighted acquisitions on large samples (up to 13 cm diameter) like human heart (3.9 cm × 8.1 cm × 7.9 cm) or hearts of large mammalian species (12 cm × 8 cm × 6 cm) by taking advantage of parallel imaging methods to reduce acquisition times. To limit the voxel averaging of the DTI metrics and depict the heterogeneity of diffusion directions, a relatively high isotropic voxel resolution of 600 µm was picked. Consequently, to maintain a reasonable acquisition time (from 9 to 12 h, depending on the matrix size), the acquisition suffered from a low angular resolution of the DW images in the six directions in comparison with recent studies in rodent species (65) or human species (66, 67).

The utility of groupwise registration and tractography for structural analysis has been massively demonstrated in neuroscience (68) and in cardiac diffusion studies (69–71), but their use remains marginal in cardiac studies and mostly nonavailable to the community. In this work, we demonstrate the use of an available open-source toolkit for both registration, tensor reorientation, and streamline generation and visualization, in the context of cardiac structure exploration that might pave the way for standardization of the cardiac diffusion postprocessing in ex vivo studies. Finally, debates about the existence of a bilayered fiber orientation need open data. A recent review by MacIver et al. (72) did a meta-analysis on ventricular architecture and refutes the presence of distinct layers. Although the proposed study has no purpose to validate the unique myocardial band theory, our results differ with the conclusion of the review and suggest the existence of the hypothetical septal myocyte angulation with a demarcation presented in the article (see Fig. 3 of the review). The mismatch with their ex vivo study could be explained by *1*) the basal location of the discontinuities that might not be visible with low-resolution DT-CMR; *2*) the size of the region of interest; at some resolution level, the bull’s eye representation hides the heterogeneity of the cardiac structure; and *3*) intraspecies variability. To support our findings and promote the reproducibility of the results, the diffusion tensor images used in the article and minimal scripts are released (see data availability).

### Limitations

The extension of these findings is, however, limited by a large number of factors including the small number of samples, the contractile state of the ex vivo sample, and the phenotypic parameters. For instance, the 3-D architecture of other mammalian species (rabbit) studied by ex vivo DTI (73) in two mechanically fixed states, approximating diastolic and systolic conditions, did not report such a pattern. More importantly, as the orientation of the fibers depends on the contraction state of the heart, the visualization of the 3-D myocardium architecture in an ex vivo sample is incomplete. Mobility studies of the fiber orientation has been recently covered in human by Moulin et al. (16) at three distinct cardiac phases (early systole, late systole, and diastasis), and limited change in transmural HA distributions were found in the inferoseptally and anteroseptally. However, the dual distribution cannot be reported at the midventricular level. One observation could explain such discrepancy: we noticed a variability in the extent of this layer near the endocardial part of the septum in the long-axis orientation. It extended from the atrioventricular (AV) node to the apical junction between the RV and LV in the sheep samples, whereas in the human sample, the layer mostly stopped after the septal attachment of the moderator band.

## DATA AVAILABILITY

Data are available at this link https://zenodo.org/record/5789035. Results are visible with the mrview viewer using the panels overlay/tensor/tractography. Command scripts that compute in native space (diffusion tensor, first eigenvectors, tractography) and command-line instructions for generating figures are also available (https://doi.org/10.5281/zenodo.6469798).

## SUPPLEMENTAL DATA

10.5281/zenodo.6469798 Supplemental Figs. S1–S3: https://doi.org/10.5281/zenodo.6469798.

## GRANTS

This work was supported by the Lefoulon Delalande foundation, National Research Agency IHU-LIRYC Grant ANR-10-IAHU04-LIRYC, and CARTLOVE Grant ANR-17-CE19-0007; partial support from the State of Upper Austria (to A. Petros); the European Union’s Horizon 2020 research and innovation program under the ERA-NET cofund action No. 680969 (ERA-CVD SIC-VALVES) (to C. M. Augustin and J. Bayer), funded by the Austrian Science Fund (FWF), Grant I 4652-B (to C. M. Augustin) and the French National Research Agency (ANR); and Grant ANR-19-ECVD-0006 (to J. Bayer).

## DISCLOSURES

No conflicts of interest, financial or otherwise, are declared by the authors.

## AUTHOR CONTRIBUTIONS

G.P., V.O., and E.V. conceived and designed research; J.M., D.E.-H., F.V., M.C., D.B., L.P., V.D., J.R., L.L., O.B., B.Q., M.H. performed experiments; J.R.-P., A.P., J.M., J.B., Y.B.-P., D.E.-H., G.R., A.N., C.M.A., M.G., G.P., V.O., and E.V. analyzed data; J.R.-P., A.P., J.M., J.B., Y.B.-P., D.E.-H., G.R., A.N., C.M.A., M.G., G.P., V.O., and E.V. interpreted results of experiments; J.R.-P., A.P., J.M., Y.B.-P., V.O., and E.V. prepared figures; J.R.-P., A.P., J.M., Y.B.-P., V.O., and E.V. drafted manuscript; J.R.-P., A.P., J.M., J.B., Y.B.-P., D.E.-H., G.R., A.N., C.M.A., F.V., M.C., D.B., L.P., V.D., J.R., L.L., O.B., B.Q., M.H., M.G., G.P., V.O., and E.V. edited and revised manuscript; J.R.-P., A.P., J.M., J.B., Y.B.-P., D.E.-H., G.R., A.N., C.M.A., F.V., M.C., D.B., L.P., V.D., J.R., L.L., O.B., B.Q., M.H., M.G., G.P., V.O., and E.V. approved final version of manuscript.
